# The Skeleton of Lateral Meningocele Syndrome

**DOI:** 10.3389/fgene.2020.620334

**Published:** 2021-01-14

**Authors:** Ernesto Canalis

**Affiliations:** Department of Orthopaedic Surgery and Medicine, UConn Musculoskeletal Institute, UConn Health, Farmington, CT, United States

**Keywords:** lateral meningocele syndrome, Lehman syndrome, genetic disorders, Notch, osteoporosis, osteoblast, osteoclast

## Abstract

Notch (Notch1 through 4) are transmembrane receptors that determine cell differentiation and function, and are activated following interactions with ligands of the Jagged and Delta-like families. Notch has been established as a signaling pathway that plays a critical role in the differentiation and function of cells of the osteoblast and osteoclast lineages as well as in skeletal development and bone remodeling. Pathogenic variants of Notch receptors and their ligands are associated with a variety of genetic disorders presenting with significant craniofacial and skeletal manifestations. Lateral Meningocele Syndrome (LMS) is a rare genetic disorder characterized by neurological manifestations, meningoceles, skeletal developmental abnormalities and bone loss. LMS is associated with NOTCH3 gain-of-function pathogenic variants. Experimental mouse models of LMS revealed that the bone loss is secondary to increased osteoclastogenesis due to enhanced expression of receptor activator of nuclear factor kappa B ligand by cells of the osteoblast lineage. There are no effective therapies for LMS. Antisense oligonucleotides targeting *Notch3* and antibodies that prevent the activation of NOTCH3 are being tested in preclinical models of the disease. In conclusion, LMS is a serious genetic disorder associated with NOTCH3 pathogenic variants. Novel experimental models have offered insight on mechanisms responsible and ways to correct the disease.

## Notch Receptors and Ligands

Notch are receptors that determine cell differentiation and function. Recent investigations have established Notch as a signaling pathway that influences the differentiation of cells of the osteoblast and osteoclast lineages and as a consequence their function and the regulation of bone remodeling (Bai et al., 2008; Engin et al., 2008; Fukushima et al., 2008; Hilton et al., 2008; Zanotti et al., 2008; Canalis et al., 2013a,b; Zanotti and Canalis, 2016). In addition to its role in skeletal homeostasis, Notch plays an important function in skeletal development.

**Figure 1:**
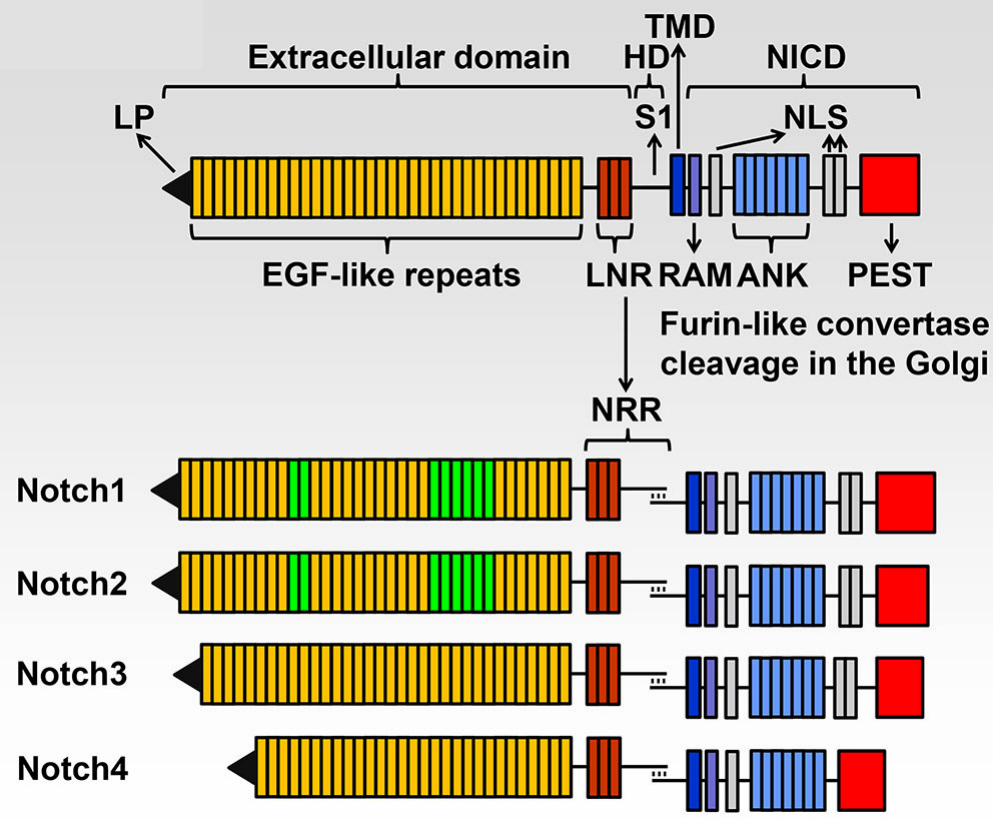
Domains of the four Notch receptors. The upper panel shows the domain and motif organization of a generic human/murine Notch receptor before cleavage at the S1 site by furin-like convertases in the Golgi compartment. The extracellular domain contains a leader peptide (LP) and multiple epidermal growth factor (EGF)-like tandem repeats followed by Lin12-Notch repeats (LNR) and the heterodimerization domain (HD). The transmembrane domain (TMD) is located between the extracellular and intracellular domains. The Notch intracellular domain (NICD) contains an RBPJκ-association module (RAM), a nuclear localization sequence (NLS), ankyrin (ANK) repeats and tandem NLS, which are followed by a proline (P)-, glutamic acid (E)-, serine (S)- and threonine (T)-rich (PEST) domain. The lower panel shows the domains and motifs of heterodimeric individual receptors, the negative regulatory region (NRR) is formed by the LNR and HD following cleavage at the S1 site. NOTCH1 and NOTCH2 have 36 EGF-like repeats; EGF 11 and 12 in green are those required for binding of NOTCH1 and NOTCH2 to JAGGED and Delta-like ligands and EGF 24-29 modulate this binding. NOTCH1 and NOTCH2 have a similar NICD, and NOTCH3 has 34 EGF-like repeats and a shorter NICD than NOTCH1 and NOTCH2. NOTCH4 has 29 EGF-like repeats and an NICD that is shorter than that of other receptors and lacks the tandem NLS located between the ANK repeats and the PEST domain. Reproduced with permission from Zanotti and Canalis (2016). The text of this figure is a word-by-word citation of the original legend.

There are four Notch (NOTCH1 through NOTCH4) receptors, and they are activated following interactions with Notch ligands, which like Notch receptors, are transmembrane proteins. These classic Notch ligands are termed JAGGED 1 (JAG1) and JAG2, and Delta-like 1 (DLL1), DLL3 and DLL4. Notch receptors have a complex structure and NOTCH1 through NOTCH4 share basic structural features (Figure 1). The extracellular domain consists of 29–36 epidermal growth factor (EGF) repeats, and EGF repeats 11 and 12 are the site of interactions of Notch with its ligands although EGF repeats 24–29 (Abruptex region) modulate the interaction at EGF repeat 11 and 12 and as a consequence Notch activation (Kelley et al., 1987; de Celis and Bray, 2000; Xu et al., 2005). The negative regulatory region (NRR) rests where the extracellular domain meets the transmembrane domain. The NRR is comprised of three Lin12-Notch repeats (LNR); they form an envelope that protects the heterodimerization domain (HD) from cleavage. This is where the cleavage required for Notch activation occurs, playing a critical regulatory role in the control of Notch signal activation (Sanchez-Irizarry et al., 2004; Gordon et al., 2007, 2009, 2015; Cordle et al., 2008). The intracellular domain of Notch (NICD) consists of seven ankyrin repeats, an RBPJκ-association module (RAM) domain and nuclear localization sequences. The NICD plays an essential function in the transcription of target genes. At the C-terminus there is a proline (P)-, glutamic acid (E)-, serine (S) - and threonine (T)-rich (PEST) domain, which is targeted for the proteasomal degradation of Notch, defining the life and duration of the Notch signal (Rogers et al., 1986; Zanotti and Canalis, 2010).

Although there are similarities among the four Notch receptors, each receptor has its own identity. Functional differences are explained by differences in the NICD interactions with recombination signal binding protein for immunoglobulin kappa J region (RBPJκ), to the temporal and specific cellular expression of the receptor and to variations in the affinity of Notch for its ligands (Wu and Bresnick, 2007; Yuan et al., 2012). Differences in the sequence of the NRR also contribute to the specific activation of each receptor. The distinct function and lack of redundancy of each Notch receptor is confirmed by the phenotype of mouse models of gene inactivation. The *Notch1* null mutation is developmentally lethal, and *Notch2* hypomorphic alleles result in perinatal death. In contrast, null mutations of *Notch3* and *Notch4* are not lethal although *Notch3* null mice have modest vascular alterations (Swiatek et al., 1994; Krebs et al., 2000, 2003; McCright et al., 2001; Domenga et al., 2004). The unique role of each Notch receptor is substantiated by the fact that genetic disorders associated with pathogenic variants of Notch receptors present with distinct phenotypic manifestations. For a detailed description of mouse models used to study the role of Notch signaling in the skeleton, the reader is referred to a recent review from the author's laboratory (Zanotti and Canalis, 2016).

NOTCH1, NOTCH2, and NOTCH3 and low levels of NOTCH4 are found in cells present in the skeleton (Bai et al., 2008; Zanotti and Canalis, 2017). NOTCH1 and NOTCH2 are expressed by osteoblasts, osteoclasts and osteocytes, whereas NOTCH3 is expressed by osteoblasts and osteocytes (Delgado-Calle et al., 2016; Zanotti and Canalis, 2017). The difference in the pattern of cellular expression confers each Notch receptor a unique function. NOTCH1 and NOTCH2 are structurally similar, whereas NOTCH3 diverges and the amino acid identity of its NICD is substantially different from that of NOTCH1 and NOTCH2 (Weinmaster et al., 1992; Swiatek et al., 1994; McCright et al., 2001). Moreover, NOTCH3 has a unique pattern of cellular expression conferring NOTCH3 a unique role in physiology (Bellavia et al., 2008). In skeletal cells, NOTCH1 inhibits the differentiation of cells of the osteoblast and osteoclast lineages. Instead, NOTCH2 enhances osteoclast differentiation by direct and indirect mechanisms (Bai et al., 2008; Engin et al., 2008; Fukushima et al., 2008; Hilton et al., 2008; Canalis et al., 2016; Yu and Canalis, 2019). NOTCH3 is preferentially expressed by vascular smooth muscle cells and cells of the osteoblast lineage, particularly osteocytes and is not expressed by myeloid cells or osteoclasts (Zanotti and Canalis, 2017). As a consequence NOTCH3 induces osteoclastogenesis by indirect mechanisms since it stimulates the expression of receptor activator of nuclear factor kappa B ligand (RANKL) by osteoblasts and osteocytes (Canalis et al., 2018). And RANKL is required for osteoclast differentiation to occur (Kong et al., 1999; Nakashima et al., 2012; Park et al., 2017).

The classic or canonical Notch ligands of the JAG and DLL families are single-pass transmembrane proteins with a conserved extracellular domain that, like Notch, contains multiple tandem EGF-like repeats. Of these ligands, JAG1 is consistently expressed by skeletal cells and its deletion phenocopies models of Notch inactivation suggesting that JAG1 is the most relevant ligand to skeletal homeostasis (Lawal et al., 2017; Zanotti and Canalis, 2017; Yu and Canalis, 2019). A variety of soluble and transmembrane proteins have been reported to interact with Notch receptors. As such, they have the potential to modify Notch effects, but should not necessarily be considered physiological activators of canonical Notch signaling. Delta-like homolog 1 (DLK-1) or PREF1 inhibits Notch activity and *Hes1* expression and Delta/Notch-like EGF-related receptor (DNER), F3 and NB3, also termed Contactin1 and Contactin6 can induce Notch signaling in neuronal cells acting through a Deltex-dependent mechanism (Hu et al., 2003; Baladron et al., 2005; Eiraku et al., 2005). Microfibril-associated glycoprotein (MAGP)1 and MAGP2 interact with the extracellular domain of Notch and induce Notch activity, but in endothelial cells MAGP1 and MAGP2 inhibit Notch signaling (Miyamoto et al., 2006; Albig et al., 2008). Studies of these non-canonical proteins interacting with Notch in skeletal cells have been restricted to nephroblastoma overexpressed, which was found to interact with Notch and reduce Notch-dependent transactivation in ST-2 stromal cells (Rydziel et al., 2007).

## Notch Signal Activation

**Figure 2:**
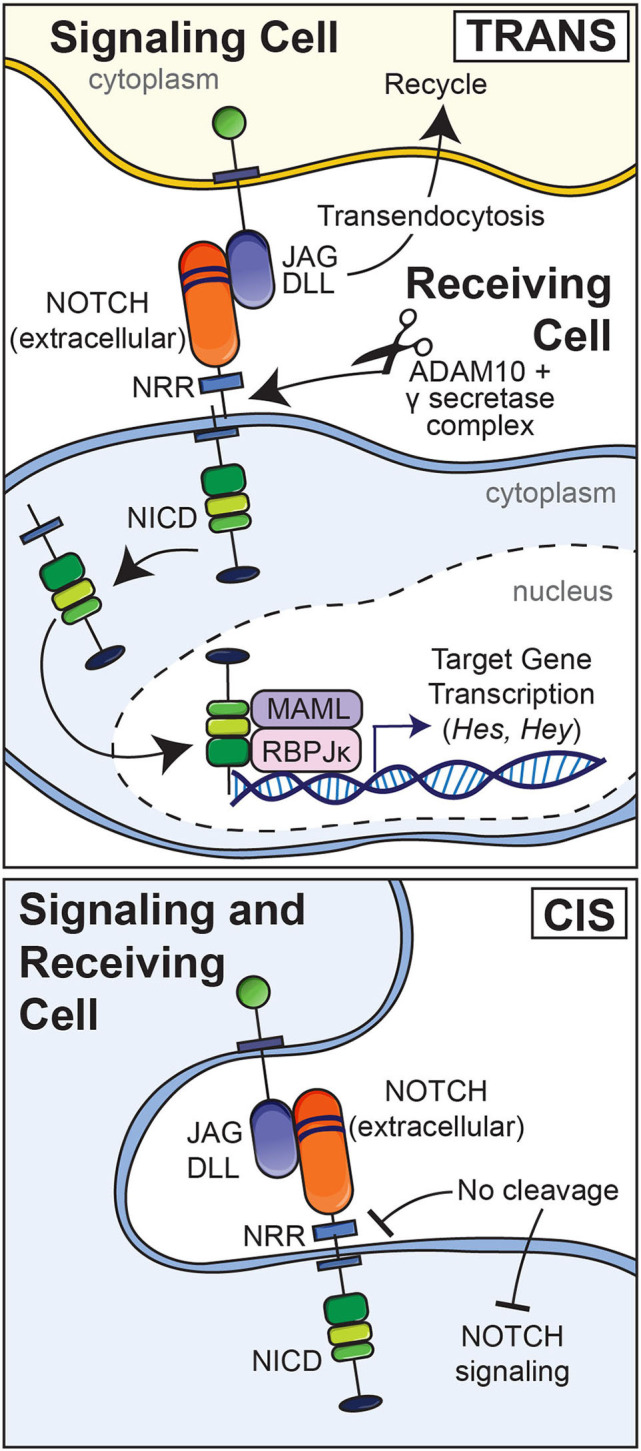
Upper panel: Notch ligands (JAG and DLL) expressed by an adjacent signaling cell bind to epidermal growth factor (EGF) repeats 11 and 12 of Notch in a receiving cell (*trans*). This results in a pulling or hinge-like effect that exposes the heterodimerization domain of Notch to the actions of the metalloprotease disintegrin and metalloprotease domain (ADAM) 10 and the γ-secretase complex. The ligand is internalized by endocytosis and recycled whereas Notch releases its intracellular domain (NICD) to the cytoplasm. The NICD translocates to the nucleus to form a complex with RBPJκ and Mastermind (MAML), and recruits co-activators to induce the transcription of target genes. Lower panel: Interactions between a Notch ligand and Notch expressed by the same cell (*cis*) result in an inhibition of Notch signaling.

Although modest levels of activation have been reported for Notch receptors under basal conditions, most Notch activation requires interactions with ligands including JAG1 and JAG2 and DLL1, DLL3, and DLL4 (Choy et al., 2017). The interaction of Notch with a ligand present in an adjacent cell results in the endocytosis of the ligand and a pulling or hinge-like effect causing the unraveling of the Lin12 repeats leaving the HD unprotected and exposed to the actions of disintegrin and metalloprotease domain-containing proteins and its subsequent cleavage by the γ-secretase complex (Figure 2) (Song et al., 1999; Ehebauer et al., 2006; Sato et al., 2007). As a result, the NICD is released into the cytoplasm and this is followed by its translocation into the nucleus. Endocytosis of the Notch ligand is followed by its recycling to the cell surface (Deblandre et al., 2001; Lai et al., 2001; Le Borgne and Schweisguth, 2003; Yamamoto et al., 2010). The consequences of the interaction of Notch with its ligands depend on whether the ligand and Notch are in the same cell (*cis*) resulting in an inhibition of activation or in a different adjacent cell (*trans*) resulting in signal activation (Figure 2). What initiates the interaction of Notch with its ligand is not known. Post-translational changes of the extracellular domain of Notch regulate Notch ligand interactions. For example, EOGT, a glycosyltransferase that transfers *N*-acetylglucosamine linked to Ser or Ther (O-GlcNAc) to specific EGF repeats of Notch, selectively enhances the binding of DLL1 and DLL4 but not that of JAG1 to Notch (Sawaguchi et al., 2017). Fringe glycosyltransferases, such as lunatic and manic fringe decrease JAG1 binding and increase DLL1 binding to Notch and radical fringe enhances the binding of both ligands to the receptor (Bruckner et al., 2000; Lei et al., 2003; LeBon et al., 2014).

The cleavage of Notch and release of the NICD to the cytoplasm and its nuclear translocation result in the formation of a complex of the NICD with RBPJκ, also termed C promoter binding factor 1 (CBF1), Suppressor of hairless, Lag1 or CSL, and with mastermind-like (MAML) (Kovall, 2007, 2008). Under basal conditions, RBPJκ is bound to DNA and associates with co-repressors to inhibit transcription. The repressors are displaced by the NICD, which also recruits activators of transcription. The NICD, RBPJκ, MAML complex induces target gene transcription (Artavanis-Tsakonas et al., 1999). Targets of the canonical Notch signaling include genes of the Hairy and enhancer of split (HES) and HES with YRPW (HEY) families of transcription factors (Ohtsuka et al., 1999; Iso et al., 2001, 2003; Katoh and Katoh, 2004). Notch signal activation is concluded following the phosphorylation of the PEST domain by cyclin-dependent kinases, resulting in the dismantling of the NICD, RBPJκ, MAML transcriptional complex, the ubiquitination of the NICD by E3 ubiquitin ligases and the ultimate degradation of the NICD (Fryer et al., 2004). Notch signaling independent of RBPJκ is considered non-canonical, a pathway poorly characterized. It is not known whether this non-canonical pathway operates in bone cells.

## Clinical Aspects of Lateral Meningocele Syndrome (LMS)

In 1977, Lehman et al. reported a child with osteosclerosis of the vault of the skull, the face and vertebrae, abnormalities of the nervous system and meninges and abnormal facial characteristics (Lehman et al., 1977). The individual presented with pronounced basilar invagination and platybasia and a myelogram revealed the presence of meningoceles. The mother shared her daughter's facial features and increased bone density of the skull. Additional cases with similar features were reported in 1978, 1995, and 1997 by Katz, Philip and Gripp and their co-workers, respectively (Katz et al., 1978; Philip et al., 1995; Gripp et al., 1997).

LMS or Lehman Syndrome (OMIM 130720), is a rare genetic disorder presenting with craniofacial anomalies, developmental delay, intellectual disability, hypotonia, decreased muscle mass, syringomyelia, Chiari Type 1 malformation, hydrocephalus and meningoceles (Gripp et al., 1997; Ejaz et al., 2016). Numerous skeletal manifestations occur including developmental defects of craniofacial structures, short stature, vertebral abnormalities characterized by kyphosis and scoliosis and hyperextensibility of small and large joints (Chen et al., 2005). The craniofacial characteristics include supraorbital ridges, down-slanted eyelid axis, ptosis, malar hypoplasia, broad nasal bridge, philtrum that is flat, undersized jaw, low set ears, high arched and cleft palate and short neck (Gripp et al., 1997; Alves et al., 2013; Correia-Sa et al., 2015). The craniofacial developmental defects include wormian bones, platybasia, and thickened calvarial vault. Pseudo clubbing of the fingers, but not acroosteolysis, is found. Cardiac valve abnormalities can be present and inner ear abnormalities and cystic kidneys were reported in one case (Cappuccio et al., 2020). Approximately 20–30 individuals with LMS have been reported. Osteoporosis is often not present in LMS possibly because most of the known cases of LMS are described in children at a young age and only occasional adults with the disease have been studied (Castori et al., 2014; Mushtaq et al., 2015). Indeed, osteoporosis was reported in a 55 year old woman affected by the disease but bone mineral density exams have not been reported in children and young adults (Castori et al., 2014). Bone biopsies have not been reported in individuals with LMS.

## Genetic Aspects of Lateral Meningocele Syndrome

Pathogenic variants of Notch receptors, Notch ligands, Notch signal modulators and components of the transcriptional complex are associated with genetic disorders affecting the skeleton. The pathogenic variants are known to cause alterations (gain- or a loss) in the function of specific Notch receptors.

The inheritance of LMS is considered to be autosomal dominant. Exome sequencing of individuals suffering from LMS revealed an association of LMS with heterozygous mutations in exon 33 of *NOTCH3* (Gripp et al., 2015). Either nonsense mutations or short deletions are found and they all cause the formation of termination codons upstream of the PEST domain. The pathogenic variants result in the translation of a truncated NOTCH3 protein product lacking the PEST domain and presumably stable. The truncated NOTCH3 NICD is capable of forming a complex with RBPJκ or CSL and MAML, and as a consequence it can regulate transcription. Since the protein is stable, signaling is prolonged resulting in a gain-of-NOTCH3 function. The diagnosis of LMS can be made by documenting the presence of truncating mutations in exon 33 of *NOTCH3* upstream of the PEST domain. The pathogenic variants associated with LMS are analogous to those reported in Hajdu Cheney Syndrome (HCS), a genetic disorder caused by pathogenic variants in exon 34 of *NOTCH2* resulting in the translation of a truncated NOTCH2 protein lacking the PEST domain and a gain-of-NOTCH2 function (Isidor et al., 2011; Simpson et al., 2011; Canalis and Zanotti, 2014; Canalis, 2018). LMS and HCS share selected clinical features, but are distinct clinical entities (Avela et al., 2011; Gripp, 2011). Indeed, some of the skeletal features of HCS such as acroosteolysis and osteoporosis with fractures are not found in LMS.

## Preclinical Models of Lateral Meningocele Syndrome

To understand the mechanisms responsible for the LMS phenotype, we created a murine model mimicking pathogenic variants discovered in individuals afflicted by LMS (Canalis et al., 2018). For this purpose, CRISPR/Cas9 technology was used to introduce a tandem termination codon in exon 33 of *Notch3* mimicking a mutation found in a subject afflicted by the disease (Gripp et al., 2015). The subject harbored a single base pair insertion at c.6692_93insC of *NOTCH3* resulting in the translation of a protein lacking the PEST domain and retaining all NICD sequences required for the formation of an active transcriptional complex. NOTCH3 DNA and amino acid sequences are conserved between human and mouse in this region, allowing the creation of a mouse model of LMS. To this end, we introduced the described human *NOTCH3* pathogenic variant into the mouse genome creating a tandem stop codon (6691-6696 ACCAAG>TAATGA), which would lead to a T2231X change at the amino acid level and a NOTCH3 protein of 2,230 amino acids (vs. 2,318 in wild types) lacking the PEST domain. The proper introduction of the *Notch3* 6691-6696 ACCAAG>TAATGA mutation was verified by DNA sequencing prior to the establishment of the mutant mouse line, which was termed *Notch3*^*em*1*Ecan*^ (synonym *Notch3*^*tm*1.1*Ecan*^).

Heterozygous mutant mice were active, mobile and no neuromuscular defects were observed. X-rays of the skull and spine were normal. Microcomputed tomography (μCT) of the distal femur demonstrated that *Notch3*^*em*1*Ecan*^ male and female mutant mice had a 35–60% decrease in cancellous bone volume and reduced connectivity density; cortical bone of mutant mice was thin and had increased porosity. Cancellous bone histomorphometry confirmed the osteopenic phenotype of *Notch3*^*em*1*Ecan*^ mice and demonstrated an increase in osteoclast number and in bone remodeling (Canalis et al., 2018).

The osteopenia of the *Notch3*^*em*1*Ecan*^ mutant mouse was secondary to an increase in the number of osteoclasts with the resulting increase in bone resorption and remodeling. The augmented number of osteoclasts was secondary to a selective increase in RANKL expression by cells of the osteoblast lineage without changes in the levels of its decoy receptor osteoprotegerin. There were no direct effects of NOTCH3 on osteoclast formation because *Notch3* is not detected in this lineage. Osteocytes, an important source of RANKL, contributed to the osteopenic phenotype of *Notch3*^*em*1*Ecan*^ mutants since *Notch3* is preferentially expressed by these cells (Nakashima et al., 2011; Xiong et al., 2011; Xiong and O'Brien, 2012). It is not established whether the induction of RANKL is mediated by Notch canonical signaling. The phenotype of *Notch3*^*em*1*Ecan*^ mutant mice is purely resorptive; osteoblast differentiation is not impaired, and bone formation was increased *in vivo* demonstrating a state of high bone remodeling. However, it is possible that supraphysiological levels of NOTCH3 NICD inhibit osteoblastogenesis as it has been reported for NOTCH1 (Zanotti et al., 2008). Although the osteopenic phenotype of *Notch3*^*em*1*Ecan*^ mice was secondary to increased RANKL, the administration of osteoprotegerin (OPG)-Fc was not considered for its correction because OPG-Fc causes osteopetrosis making the interpretation of the results difficult or not possible (Bargman et al., 2012).

To ensure that non-skeletal tissues were not affected by the mutation, we documented no abnormalities by histopathology of the heart, liver, spleen, lungs, brain and kidneys of *Notch3*^*em*1*Ecan*^ mutant mice. We also documented that the pattern of *Notch3* mRNA expression was not different between mutant and wild type mice in the tissues examined. Moreover, serum parathyroid hormone and estrogen levels were not different between *Notch3*^*em*1*Ecan*^ mice and control mice. Fasting glucose and insulin levels, lean body and fat tissue mass were not different between *Notch3*^*em*1*Ecan*^ and control mice. These results suggest that the *Notch3*^*em*1*Ecan*^ phenotype is due to direct effects of NOTCH3 on the skeleton and not to a secondary effect in non-skeletal tissues or concurrent neomorphic activities. The expression of the Notch target genes *Hey1, Hey2* and *HeyL* was increased in cells from *Notch3*^*em*1*Ecan*^ mice indicating that Notch signal activation was enhanced.

The phenotype of the heterozygous *Notch3*^*em*1*Ecan*^ mutant mouse recapitulates limited aspects of the LMS phenotype in humans, and mutant mice do not manifest the array of neurological and developmental abnormalities observed in the human syndrome. It is important to note that we were unable to generate homozygous mutant mice following heterozygous intercrosses and we suspect embryonic lethality due to developmental defects. It is conceivable that homozygous mutant *Notch3*^*em*1*Ecan*^ mice had additional phenotypic manifestations in line with the manifestations of the human disease.

## Interventions to Ameliorate the Lateral Meningocele Syndrome Phenotype

Currently, there are no therapeutic interventions to ameliorate the LMS phenotype other than the surgical interventions aimed at correcting or ameliorating selected neurological and skeletal defects (Brown et al., 2017; Cuoco et al., 2020). A central problem with LMS is the genetic nature of the disease and the fact that the phenotype becomes established during embryogenesis with multiple organs affected at birth. Moreover, there is no practical or effective intervention that corrects genetic abnormalities associated with LMS, resulting in the unsuccessful management of individuals afflicted by this as well as by many other genetic disorders. Gene editing has been proposed to correct mutations in mice and humans (Savic and Schwank, 2016; Porteus, 2019). However, for gene editing to be effective ideally a pathogenic variant should be edited and repaired in the germline making the approach not practical or readily available for therapeutic intervention (Nelson et al., 2016; Tabebordbar et al., 2016). Moreover, ethical concerns have been raised regarding genome editing in human embryos (Savic and Schwank, 2016; Daley et al., 2019). A specific tissue could be targeted with vectors with preferential affinity for the tissue affected or with constructs driving enzymes, such as Cas9, using tissue-specific promoters to cut and replace the mutant DNA with repair DNA (Long et al., 2016). However, it is quite challenging to introduce a single-stranded DNA fragment into the cell nucleus to repair the double-stranded DNA break prior to non-homologous end-joining without the introduction of insertions/deletions (indels). The problem is complicated further in phenotypes manifested in the heterozygous state, such as LMS, since Cas9 creates a break in the mutant as well as in the wild type allele with the potential of additional indels and unpredicted homozygous mutations unless both alleles are properly repaired.

The administration of anti-sense oligonucleotides (ASO) is a novel intervention used to downregulate wild type as well as mutant transcripts. ASOs have been utilized to silence mutant genes in the central nervous system, retina and liver (Carroll et al., 2011; Limmroth et al., 2014; Murray et al., 2015; McCampbell et al., 2018; Shy, 2018; Zhao et al., 2018; Zhu et al., 2018). ASOs are single-stranded synthetic nucleic acids that bind to target mRNA by Watson-Crick pairing and result in the degradation of mRNA by RNase H (Cerritelli and Crouch, 2009; Bennett et al., 2017). The transport of ASOs to bone has required delivery systems to target ASOs to the skeleton and the technology has not been applied for the correction of gene mutations in this organ (Zhang et al., 2012). It is possible that the pathogenic variant associated with Lehman Syndrome can be targeted to ameliorate the phenotype associated with the NOTCH3 gain-of-function using an ASO approach. The ASO strategy was recently tested in an experimental mouse model of HCS termed *Notch2*^*tm*1.1*Ecan*^ and characterized by a NOTCH2 gain-of-function and severe osteopenia secondary to increased osteoclastogenesis (Canalis et al., 2020). Delivery of Notch2 ASOs *in vitro* decreased *Notch2* wild type and mutant expression, and NOTCH2 activation, and inhibited osteoclastogenesis in *Notch2*^*tm*1.1*Ecan*^ osteoclast precursors. Importantly, the administration of Notch2 ASOs *in vivo* ameliorated the osteopenia of HCS *Notch2*^*tm*1.1*Ecan*^ heterozygous mice.

Similar work is in progress in *Notch3*^*em*1*Ecan*^ heterozygous mutant mice to test whether ASOs targeting either *Notch3* or preferably the *Notch3* mutation improve the phenotypic manifestations of this experimental model of LMS. The targeting of the *Notch3* mutant allele with ASOs to downregulate the allele specifically would create a wild type heterozygous state. The approach could be used as a therapeutic intervention not only in Lehman Syndrome but also in other dominant monogenic disorders of the skeleton. Humans with homozygous *NOTCH3* loss-of-function pathogenic variants exhibit vascular manifestations reminiscent of cerebral autosomal dominant arteriopathy with subcortical infarcts and encephalopathy (CADASIL) (Pippucci et al., 2015). Therefore, the selective downregulation of the mutant allele would be a preferable approach by avoiding the generalized downregulation of *NOTCH3*.

The creation of a mutant mouse replicating the genetic alteration found in LMS or Lehman Syndrome allowed us to test whether the skeletal phenotype of *Notch3*^*em*1*Ecan*^ mice could be reversed by preventing the activation of NOTCH3 with anti-NOTCH3 antibodies targeting the NRR, the site required for the cleavage and activation of NOTCH3. Anti-NOTCH3 NRR antibodies decreased Notch activation and the expression of RANKL by osteoblasts, and as a consequence reversed the cancellous bone osteopenia of *Notch3*^*em*1*Ecan*^ mutant mice (Yu et al., 2020). Although the approach demonstrated the effectiveness of anti-NOTCH3 NRR antibodies in downregulating Notch signaling and reversing the skeletal phenotype, a limitation of their use is the fact that they block both NOTCH3 mutant as well as NOTCH3 wild type activation leading to a generalized NOTCH3 knockdown and the potential of unwanted collateral effects.

**Figure 3:**
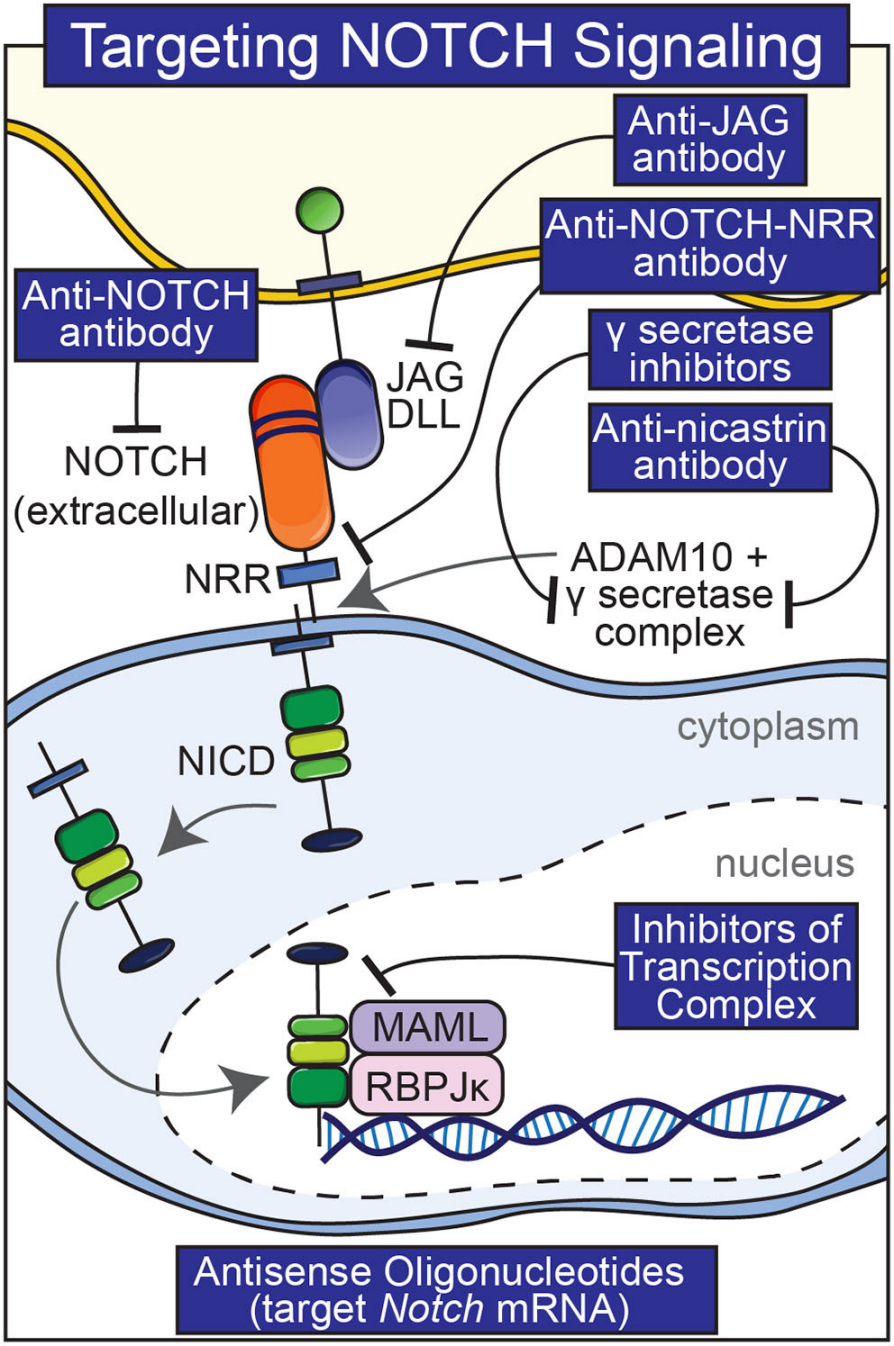
Pharmacological interventions to temper Notch signaling. Antibodies to various components of Notch signaling (Notch, Notch NRR, JAG, nicastrin), inhibitors of the γ-secretase complex, and small peptides that preclude the formation of a Notch NICD, RBPJκ, MAMAL complex are shown.

Pharmacological interventions other than anti-Notch antibodies have been used to manipulate Notch signaling in skeletal and non-skeletal cells although they suffer from greater shortcomings. These interventions include the use of biochemical inhibitors of Notch activation, antibodies to various components of the Notch signaling pathway and molecules that interfere with the formation of an NICD/RBPJκ/MAML complex (Figure 3) (Ryeom, 2011). γ-secretase inhibitors are frequently used to block the cleavage of the Notch receptor (De Strooper et al., 1999). It is important to note that these inhibitors are not specific and the substrates of the γ-secretase complex are many (Duggan and McCarthy, 2016). Because nicastrin forms part of the γ-secretase complex, anti-nicastrin antibodies can be used as an alternative (Siebel and Lendahl, 2017). Thapsigargin prevents the maturation and folding of Notch and has been used to inhibit the effects of Notch (Ilagan and Kopan, 2013). A limitation of thapsigargin, anti-nicastrin antibodies and γ-secretase inhibitors is that they inhibit all Notch receptors, and their long-term use would result in an indiscriminate knock-down of Notch activation and possible unwanted effects. Generalized knockdown of Notch signaling can result in significant gastrointestinal toxicity and diarrhea due to the fact that Notch directs intestinal precursor cells toward an epithelial cell fate and away from secretory cell differentiation (van Es et al., 2005; Zecchini et al., 2005; Garber, 2007). Molecules that interfere with the formation of an active NICD/RBPJκ/MAML complex have been reported to inhibit Notch isoforms; their efficacy is not fully established (Moellering et al., 2009).

## Major Gaps in Knowledge and Future Studies

A limitation of the *Notch3*^*em*1*Ecan*^ mutant mouse model is that it replicates the human disease partially and future mutant mouse models should be developed to have a greater understanding of mechanisms responsible for the manifestations of LMS. However, the availability of the *Notch3*^*em*1*Ecan*^ model has advanced our knowledge of the disease and implies that the osteopenic phenotype is due to an induction of RANKL by cells of the osteoblast lineage. This could be verified by the use of conditional mouse models, that would allow the introduction of the mutation in this lineage. Conditional models also could serve to determine the contributions of vascular NOTCH3 to the skeletal phenotype. This is particularly important since *Notch3* is preferentially expressed by smooth muscle vascular cells and *NOTCH3* pathogenic variants are associated with CADASIL (Chabriat et al., 2009; Gridley, 2010; Filipowska et al., 2017; Watson and Adams, 2018).

To ensure that the *Notch3*^*em*1*Ecan*^ phenotype is due to a gain-of-NOTCH3 function and not due to neomorphic manifestations of the mutation, it would be worthwhile comparing the phenotypes of the *Notch3*^*em*1*Ecan*^ mouse model to models of wild type NOTCH3 overexpression (Lafkas et al., 2013). However, these alternate models could result in supraphysiological concentrations of NOTCH3, so that comparisons need to be interpreted with caution. There is limited information about the physiological role of NOTCH3 in the skeleton since skeletal phenotypes of *Notch3* null mice have not been published. Similarly, previous work on *Notch3*^*em*1*Ecan*^ mice has been limited to the description of postnatal skeletal phenotypes and there is no information on developmental phenotypes, particularly in homozygous mutant mice due to developmental lethality. This could also be the case in humans since only heterozygous pathogenic variants have been reported in LMS. Future studies in preclinical mouse models should serve to define the role of NOTCH3 in skeletal physiology and the contributions of vascular NOTCH3 to bone homeostasis.

Current therapies for LMS have been palliative at best and future work should be directed to the development of effective therapies aimed at correcting or silencing the pathogenic variant allele. However, a number of technical and ethical issues need to be resolved before these approaches become a reality.

## Conclusions

In conclusion, LMS or Lehman Syndrome is a rare disorder associated with pathogenic variants in exon 33 of *NOTCH3*. LMS is characterized by neurological, craniofacial and skeletal developmental defects. Preclinical mouse models have served to enhance our understanding of the pathogenesis of the disease and mechanisms responsible for the skeletal phenotype and ways to test possible treatment interventions.

## Author Contributions

EC: conceptualization, writing, editing, and funding acquisition.

## Conflict of Interest

The author declares that the research was conducted in the absence of any commercial or financial relationships that could be construed as a potential conflict of interest.
